# Simulation study towards quantitative X-ray and neutron tensor tomography regarding the validity of linear approximations of dark-field anisotropy

**DOI:** 10.1038/s41598-021-97389-y

**Published:** 2021-09-16

**Authors:** Jonas Graetz

**Affiliations:** 1 Universität Würzburg, Lehrstuhl für Röntgenmikroskopie, Würzburg, Germany; 2 Fraunhofer IIS, Magentic Resonance and X-ray Imaging Department, Würzburg, Germany

**Keywords:** Imaging techniques, Imaging techniques, Applied mathematics, Applied physics

## Abstract

Tensor tomography is fundamentally based on the assumption of a both anisotropic and linear contrast mechanism. While the X-ray or neutron dark-field contrast obtained with Talbot(-Lau) interferometers features the required anisotropy, a preceding detailed study of dark-field signal origination however found its specific orientation dependence to be a non-linear function of the underlying anisotropic mass distribution and its orientation, especially challenging the common assumption that dark-field signals are describable by a function over the unit sphere. Here, two approximative linear tensor models with reduced orientation dependence are investigated in a simulation study with regard to their applicability to grating based X-ray or neutron dark-field tensor tomography. By systematically simulating and reconstructing a large sample of isolated volume elements covering the full range of feasible anisotropies and orientations, direct correspondences are drawn between the respective tensors characterizing the physically based dark-field model used for signal synthesization and the mathematically motivated simplified models used for reconstruction. The anisotropy of freely rotating volume elements is thereby confirmed to be, for practical reconstruction purposes, approximable both as a function of the optical axis’ orientation or as a function of the interferometer’s grating orientation. The eigenvalues of the surrogate models’ tensors are found to exhibit fuzzy, yet almost linear relations to those of the synthesization model. Dominant orientations are found to be recoverable with a margin of error on the order of magnitude of 1$^{\circ }$. Although the input data must adequately address the full orientation dependence of dark-field anisotropy, the present results clearly support the general feasibility of quantitative X-ray dark-field tensor tomography within an inherent yet acceptable statistical margin of uncertainty.

## Introduction

The anisotropic nature of X-ray and neutron dark-field contrast allows imaging of directional information within unresolved substructure of a sample and thereby provides the fundamental prerequisite to tensor-valued volume imaging analog to other anisotropic contrast modalities e.g. in the field of magnetic resonance imaging. Following on demonstrations of planar dark-field anisotropy e.g. by Jensen et al.^1^, extensions of anisotropic dark-field imaging to non-scalar volume reconstruction techniques have been shown by Malecki et al., Bayer et al., Vogel et al., Wieczorek et al., Dittmann et al., Gao et al., Kim et al.^2–8^. Common to all of these approaches is a heuristic 3D extension of planar dark-field anisotropy as a function of the interferometer sensitivity axis orientation (as is e.g. explicitly exploited in Schaff et al.^9^ to decouple the problems of volume and tensor reconstruction), while the specific signal models and reconstruction algorithms vary considerably. Malecki et al.^2^ and Vogel et al.^4^ employed a temporary expansion of voxels into a non-orthogonal basis of principal scattering orientations, with subsequent fitting to a non-linear tensor model. Wieczorek et al.^5^ proposed an expansion into spherical harmonics (which represent an orthogonal expansion basis for functions over the unit sphere), and Bayer et al.^3^ used a sinusoidal model describing the anisotropy of each voxel only within the tomographic plane. The expansion basis proposed by Malecki et al.^2^ was also adopted by Schaff et al.^9^ and Kim et al.^8^. Dittmann et al.^6^ and Gao et al.^7^ proposed the use of linear rank two tensor models for the description of voxel anisotropy analog to other fields of tensor tomography (e.g., diffusion tensor imaging in the context of magnetic resonance tomography).

Although formal extensions of the Radon transform and its inverse to vector and tensor fields (c.f. e.g. the overview given by Defrise and Gullberg^10^) establish the theoretic feasibility of non-scalar tomography, the linear projection models assumed and required within the mathematical conception of tensor tomography are not actually well reproduced by the available physical contrast modality (dark-field). In a recent review, the origination and actual orientation dependence of dark-field contrast for anisotropic mass distributions has been investigated in detail (Graetz et al.^11^), yielding a minimal yet non-linear model capturing the central features of general dark-field anisotropy including in particular also the effect of varying scattering cross section in addition to the characteristic dependence of dark-field contrast on a structure’s correlation lengths. I.e., general dark-field anisotropy is concluded to be a function of two orientations (the optical axis and a perpendicular axis of interferometer sensitivity), which has not been taken into account so far and in particular challenges the general assumption in present literature that dark-field signals may be described as a function over the unit sphere.

While this additional complexity, and the non-linearity in particular, are highly undesirable with regard to tomographic reconstruction, the derived physically based model allows to synthesize large amounts of anisotropic dark-field signals that will here be used to systematically study the general applicability of approximative linear tensor models suited for classic tensor tomography. As it likewise uses a tensor to parametrize the structural anisotropy of the considered volume element, direct comparisons between the respective tensors of the physically motivated non-linear signal model and the mathematically motivated linear tensor models can be made. Especially the qualitative scaling behavior of the reconstructed eigenvalues with respect to the extents of the original mass distribution as well as the intrinsic uncertainty in the reconstruction of orientation vectors are of concern here, while further results on the root mean square errors of the simplified models provide additional qualitative insights. The analyses form the basis towards a more detailed and quantitative understanding of X-ray or neutron dark-field tensor tomography.

## Methods

### Physical model of dark-field anisotropy

Dark-field contrast originates from a sample’s microscopic density variations below the imaging resolution, which may be described using auxiliary functions reproducing the typical densities and auto-correlation properties. This was e.g. proposed by Yashiro et al.^12^ and Lynch et al.^13^ in the context of their wave optical derivations of dark-field origination, and is also common in other theories of scattering. Based on these concepts, a minimal physically motivated model of dark-field orientation dependence for the case of arbitrary sample rotations (as opposed to rotations only about the optical axis) has been derived in Graetz et al.^11^. The central results shall be briefly recapitulated here.

Anisotropy within a considered volume element is modeled by means of a three dimensional Gaussian mass density distribution $\rho (\vec {r})$, which is formulated as

1 $$\begin{aligned} \rho (\vec {r})\propto \exp \left(-\frac{1}{2}\vec {r}\,\varvec{T}\,\vec {r} \right) \end{aligned}$$

and parametrized by a spatial coordinate $\vec {r}\in \mathbb {R}^{3}$ and a positive definite $3\times 3$ tensor $\varvec{T}\in \mathbb {R}^{3\times 3}$

2 $$\begin{aligned} \varvec{T}=\left[ \begin{array}{ccc} T_{\mathrm {xx}} &{} T_{\mathrm {xy}} &{} T_{\mathrm {xz}}\\ T_{\mathrm {xy}} &{} T_{\mathrm {xx}} &{} T_{\mathrm {yz}}\\ T_{\mathrm {xz}} &{} T_{\mathrm {yz}} &{} T_{\mathrm {zz}} \end{array}\right] =\varvec{R}\left[ \begin{array}{ccc} \sigma _{1}^{-2} &{} 0 &{} 0\\ 0 &{} \sigma _{2}^{-2} &{} 0\\ 0 &{} 0 &{} \sigma _{3}^{-2} \end{array}\right] \varvec{R}^{T}\,, \end{aligned}$$

with $\sigma _{i}\in \mathbb {R}_{>0}$ denoting the Gaussian ellipsoid’s standard deviations along its principal axes and $\varvec{R}$ being a unitary rotation matrix characterizing its orientation. As an auxiliary description of the substructure of individual volume elements within the field of view of an imaging system, the spatial scale of $\rho (\vec {r})$ is assumed to range below the system’s voxel size. Typical structure scales giving rise to X-ray dark-field contrast range between $10^{-6}\,\mathrm {m}$ and $10^{-4}\,\mathrm {m}$, whereas pixel or voxel sizes commonly range on the order of magnitude of $10^{-4}\,\mathrm {m}$. The specific absolute scales are however of no further relevance to the present considerations.

**Figure 1:**
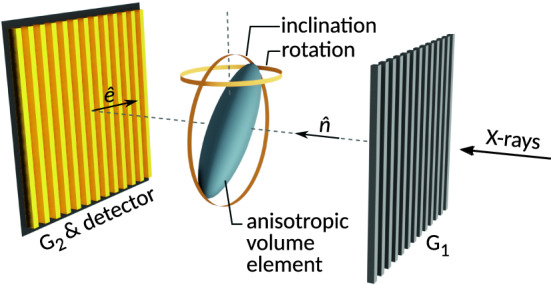
Sketch of a Talbot interferometer imaging an anisotropic volume element. From left to right: Integrating detector pixel with analyzer grating G2, volume element with indicators for the rotations and inclinations referred to in Fig. 2, and the modulating grating G1. The optical axis and orientation of interferometer sensitivity are indicated by $\hat{n}$ and $\hat{e}$ respectively. (Figure adapted from Graetz et al.^11^).

By projecting the 3D Gaussian mass density distribution along the imaging system’s optical axis $\hat{n}$ (cf. Fig. 1) and evaluating both its resulting mean variance $\sigma _{\Phi }^{2}$ over a fixed area (typically, the pixel size) and its auto-correlation function $\gamma (\xi )$ along the axis of interferometer sensitivity $\hat{e}$, the following minimal model of dark-field orientation dependence has been, based on concepts introduced in different ways by Yashiro et al.^12^ and Lynch et al.^13^, derived in Graetz et al.^11^:

3 $$\begin{aligned} \mu _{\mathrm {DF}}(\varvec{T})=-\ln (v)&\propto \frac{1}{\sqrt{T_{\mathrm {zz}}}} \left(T_{\mathrm {xx}} -\frac{T_{\mathrm {xz}}^{2}}{T_{\mathrm {zz}}}\right) \nonumber \\ \text {for}\quad \det (\varvec{T})=\prod _{i}\sigma _{i}^{-2}&=\mathrm {const.} \nonumber \\ \text {and}\quad {\hat{n}}&=(0,0,1)\nonumber \\ \hat{e}&=(1,0,0) \end{aligned}$$

with $v\in [0,1]$ denoting the sample-induced visibility loss measurable with grating interferometers. Given the purpose of Eq. (3) to capture the essential interrelations of scattering cross section and auto-correlation width of a rotating anisotropic scatterer, a number of leading order approximations are involved in the derivation of Eq. (3), for the details of which the reader shall be referred to Graetz et al.^11^. In particular, any rotation invariant factors affecting only the global scale of $\mu _{\mathrm {DF}}$ have been dropped. The approach is finally justified by the consistency of Eq. (3) with actual experiments^11^.

$\varvec{T}$ is without loss of generality evaluated in the coordinate system of the grating interferometer (in which $\hat{n}$ and $\hat{e}$ align with the *z*- and *x*-axis respectively). General orientations of the acquisition system characterized by $\hat{n}$ and $\hat{e}$ other than (0, 0, 1) and (1, 0, 0) can be considered by a respective (inverse) rotation transformation applied to $\varvec{T}$ prior to evaluating Eq. (3).

The prefactor $T_{\mathrm {zz}}^{-1/2}$ corresponds to the mass distribution’s standard deviation (i.e., its extent) along the optical axis and is proportional to the leading order of the scattering cross section, while the term $(T_{\mathrm {xx}}-\frac{T_{\mathrm {xz}}^{2}}{T_{\mathrm {zz}}})$ corresponds to the inverse variance of the projected Gaussian density distribution along $\hat{e}$ and results from its auto-correlation function. Whenever the modeled ellipsoid is not inclined with respect to the optical axis, the off-diagonal term $T_{\mathrm {xz}}$ becomes 0. This is typically the case in anisotropic dark-field imaging of planar objects, which are aligned parallel to the gratings and rotated only about the optical axis. Likewise, off-diagonal terms vanish in the isotropic case, i.e., when $\sigma _{1}=\sigma _{2}=\sigma _{3}$. Any terms affecting only the absolute scale of $\mu _{\mathrm {DF}}$ have been neglected as solely the orientation dependence shall be studied (note e.g. a global scaling with $\det (\varvec{T})^{-1/2}$, the material’s refractive index at a given X-ray or neutron energy, and the specific correlation distance of the instrument, cf.^11^).

**Figure 2:**
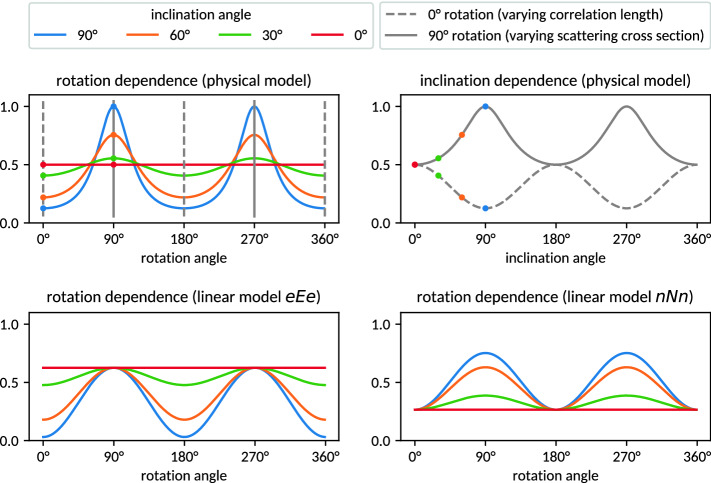
The different orientation dependencies of the physical model of dark-field anisotropy and the linear tensor models considered for reconstruction. Modeled is an elongated object inclined with respect to a rotational axis perpendicular to both the optical axis and the axis of interferometer sensitivity, cf. Fig. 1. The original signal (upper left) is generated according to Eq. (3) (with $\sigma _{3}/\sigma _{1}=\sigma _{3}/\sigma _{2}=2$) and arbitrarily scaled to 1. The respective scales and offsets of the linear approximations (bottom row) emerge from the reconstruction procedure.

Figure 2 (upper left) gives an example of anisotropic dark-field contrast according to Eq. (3) for an elongated structure inclined with respect to the optical axis. The corresponding experimental configuration is illustrated in Fig. 1. Figure 2 (upper right) illustrates the special cases of either constant scattering cross section (dashed) or constant auto-correlation width (solid). In the former case, dark-field anisotropy arises solely from variations of the sample’s auto-correlation width along $\hat{e}$, in the latter case solely from variations in scattering cross section along $\hat{n}$. While classic anisotropic dark-field imaging of planar samples perpendicular to the optical axis needs to consider only the former case, both effects need to be considered in the general case of tomographic imaging.

Equation 3 will be used here to synthesize dark-field signals required for the systematic evaluation of tensor reconstruction techniques based on the mathematically motivated linear signal models addressed in the following Section. Figures 1, 2 give an illustration, which will be discussed in the following.

### Linear tensor models and iterative reconstruction

A fundamental assumption for volume reconstruction from projections is the linearity of the projection process. I.e., the sum of projections of individual volume elements is expected to be equivalent to the projection of the sum of the respective volume elements. In classic X-ray tomography, this is ensured both by Beer’s law of attenuation and the assumption of an isotropic contrast mechanism. Equation 3 however is clearly non-linear with respect to the tensor $\varvec{T}$ characterizing individual volume elements, and should, with regard to tomographic volume reconstruction, be replaced by a suitable linear surrogate.

Classic tensor tomography considers models of the form $\hat{r}\varvec{U}\hat{r}$ comprising a unit orientation vector $\hat{r}\in \mathbb {R}^{3}$ with $\left\| \hat{r}\right\| =1$ and a symmetric tensor $\varvec{U}\in \mathbb {R}^{3\times 3}$ with $\varvec{U}=\varvec{U}^{T}$, which are generally linear (i.e., $\sum _{j}\hat{r}\,\varvec{U}^{(j)}\hat{r}=\hat{r}(\sum _{j}\varvec{U}^{(j)})\hat{r}\,$). Given the actual dark-field signal dependence on both the optical axis $\hat{n}$ and the direction $\hat{e}$ of grating sensitivity, two obvious models thus suggest themselves:

4 $$\begin{aligned} \mu _{\mathrm {DF}}(\hat{e},\hat{n},\varvec{N})\approx \hat{n}\varvec{N}\hat{n} \end{aligned}$$

and

5 $$\begin{aligned} \mu _{\mathrm {DF}}(\hat{e},\hat{n},\varvec{E})\approx \hat{e}\varvec{E}\hat{e}\,, \end{aligned}$$

which capture either of the dark-field contrast’s orientation dependencies respectively. The designations $\varvec{N}$ and $\varvec{E}$ for the respective symmetric $3\times 3$ tensors have been chosen for clear discernibility of both models. They both do represent gross simplifications whose adequacy remains to be justified, and it therefore is the aim of the present article to discuss their relation to the actual contrast mechanism of dark-field imaging better described by Eq. (3).

To this end, tensors $\varvec{N}$ and $\varvec{E}$ will be reconstructed from a set of scalar dark-field projections $\mu _{\mathrm {DF}}^{(i)}$ (enumerated by *i*) synthesized with Eq. (3) from a given mass distribution tensor $\varvec{T}$ and acquisition geometry (characterized by a set of acquisition system orientations $\hat{n}^{(i)},\hat{e}^{(i)}$), which will be detailed in Sect. 2.3. The following iterative scheme as proposed by the author in^6^ will be used for the reconstruction of $\varvec{N}$ and $\varvec{E}$ from projection data respectively:

6 $$\begin{aligned} U_{mn}^{(k)}&=U_{mn}^{(k-1)}+\lambda _{k}\overbrace{r_{m}^{(i_{k})}r_{n}^{(i_{k})}\smash [b]{(\underbrace{\mu _{\mathrm {DF}}^{(i_{k})}-\sum _{mn}r_{m}^{(i_{k})}U_{mn}^{(k-1)}r_{n}^{(i_{k})}}_{\text {residual}})}}^{\text {back projection}} \nonumber \\ \text {with}\quad U_{mn}^{(0)}&=0\nonumber \\ \text {and}\quad {\lambda _{k}}&=\lambda _{0}2^{-k/\tau },\quad \lambda _{0}\in \,]0,1]\nonumber \\ \text {assuming}\quad {\bigl \Vert \hat{r}\,\bigr \Vert }&=1, \end{aligned}$$

with *k* enumerating iterations, $i_{k}$ denoting the particular projection *i* considered at iteration *k*, and $\lambda _{k}$ being a relaxation factor damping convergence. The specific back projection procedure within Eq. (6) can be verified to have two important properties: changes to $U_{mn}^{(k)}$ will scale with the residual error between data and model, and the update is performed by means of a pseudo-inverse of the linear tensor model $\hat{r}\varvec{U}\hat{r}$ such that immediate consistency of $\hat{r}\,\varvec{U}^{(k)}\hat{r}$ with the considered projection $\mu _{\mathrm {DF}}^{(i_{k})}$ is achieved for $\lambda _{k}=1$. The continuous reduction of $\lambda _{k}$ accounts for the expected inconsistency between $\mu _{\mathrm {DF}}$ and the linear tensor models by gradually reducing the impact of individual projections $\mu _{\mathrm {DF}}^{(i)}$ and thus their specific order of consideration as convergence to a mean solution progresses.

In total, each individual projection will here be considered 25 times throughout the iterative process, with $\lambda _{0}=.2$, $\tau =3k_{\max }/25$, and $k_{\max }$ being 25 times the number of total projections $\mu _{\mathrm {DF}}^{(i)}$ (cf. following Section).

**Figure 3:**
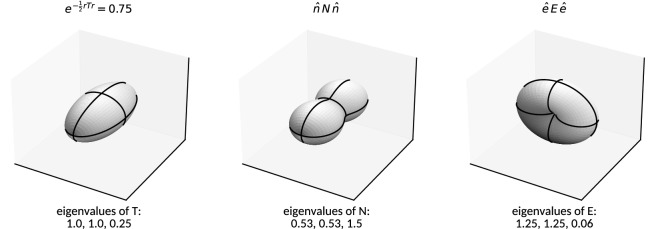
Illustrations of the considered tensors and signal models: On the left, an anisotropic Gaussian mass density distribution described by $\varvec{T}$ (with $\sigma _{3}/\sigma _{1}=\sigma _{3}/\sigma _{2}=2$) is represented by an iso-surface. The resulting dark-field signal cannot be represented in three dimensions given its dependence on two orientation vectors. On the center and right, the reconstructed signal approximations parametrized by either of the two orientations are shown. Both approximate models align with the principal orientation of the original mass distribution. Systematic comparisons of eigenvectors and eigenvalues are shown in Figs. 6, 7, 8.

Figure 2 gives an example comparison of the simplified linear signal models (bottom row) with the original dark-field signal (top row) used as input to the reconstruction procedure. The modeling of only either of both orientation dependencies manifests itself at 0$^{\circ }$ (180$^{\circ }$) and 90$^{\circ }$ (270$^{\circ }$) respectively. A spatial representation of the reconstructed signal models and comparison to the original mass distribution is given in Fig. 3, illustrating the ability to reproduce the orientation of the underlying structure.

### Projection geometry

**Figure 4:**
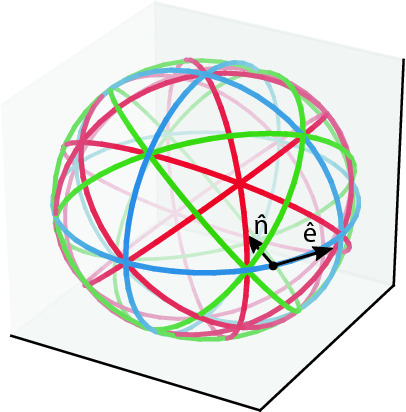
Circular scanning trajectories (expressed in the sample coordinate system) about a volume element in the center of the circumscribed sphere. Each trajectory describes a set of projection directions $\hat{n}$ pointing towards the center and associated tangential orientations of interferometer sensitivity $\hat{e}$. For better visualization, they have been grouped into trajectories about the coordinate axes (blue), about their face diagonals (red) and about their space diagonals (green). Intersections of trajectories represent points of constant projection direction $\hat{n}$ at varying dark-field sensing directions $\hat{e}$. For the present study, each trajectory is sampled at 29 equidistant points, yielding a total of 377 distinct combinations $(\hat{n}^{(i)}, {{\hat{e}}}^{(i)})$ enumerated by *i*.

The reconstruction of individual tensor voxels from scalar projections requires a number of such projections sufficiently covering the parameter space of the signal model, i.e., sufficiently covering variations in both orientations $\hat{n}$ and $\hat{e}$. Figure 4 depicts circular trajectories about a volume element in the origin. Each point on the trajectories represents a different orientation both of the optical axis $\hat{n}$ (direction of projection, pointing towards the center), and the respective orientation $\hat{e}$ of grating sensitivity (here tangential to the respective circular rotation orbits). Each trajectory is normal to one of the coordinate axes (blue) or to one of their diagonals (red and green), yielding a total of 13. Such a geometry is, in practice, realized by placing a sample in different orientations on the rotary stage of a typical tomography setup with the grating bars of the Talbot interferometer running parallel to the rotational axis. The circular trajectories will be sampled at discrete, equidistant points. With regard to the present simulations of individual volume elements, each trajectory is sampled at 29 points equidistantly covering the full circle.

### Parameter exploration

The parameter space of the physical dark-field model given in Eq. (3) consists of the to-be-reconstructed tensor characterizing the volume element on the one hand and the orientations $\hat{n}$ and $\hat{e}$ of the considered projection on the other hand. While the coverage of projection parameters, which correspond to the imaging procedure, has just been discussed, an adequate exploration scheme for the space of anisotropic volume elements described by $\varvec{T}$ shall be addressed here.

At first it can be confirmed that the absolute scale of $\varvec{T}$ does not affect the orientation dependence of $\mu _{\mathrm {DF}}$ (see Eq. 3). It is therefore sufficient, without loss of generality, to require $\text {trace}(\varvec{T})=1=\sum _{i}\sigma _{i}^{-2}$ for the present purposes, thereby implicitly constraining one eigenvalue and reducing the parameter space to the remaining two. Further, permutations of equivalent sets of eigenvalues correspond to rotations, which are treated separately. Based on these considerations, the following definitions can be made with regard to an exhaustive coverage of the parameter space:

7 $$\begin{aligned} \begin{aligned} \sigma _{1}^{-2}&\in [0,1/3]\\ \sigma _{2}^{-2}&\in [0,1/2]\\ \sigma _{3}^{-2}&\in [1/3,1]\\ \text {with}\quad {\sigma _{1}^{-2}}&\le \sigma _{2}^{-2}\le \sigma _{3}^{-2}\\ \text {and}\quad \sum _{i}\sigma _{i}^{-2}&=1\,. \end{aligned} \end{aligned}$$

**Figure 5:**
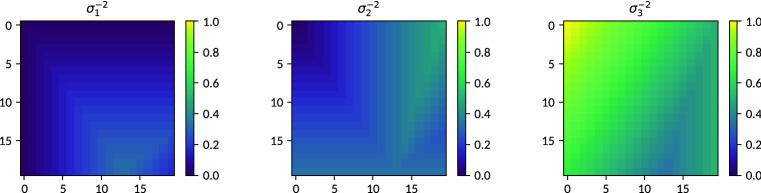
Coverage of the parameter space (on a 20${}\times {}$20 grid) of possible eigenvalue combinations in $\varvec{T}$ according to Eq. (7). The salient triangular region extending from the top right corner to the bottom (cf. center and right panels) corresponds to redundancies in the coverage of the range $\sigma _{2}^{-2},\sigma _{3}^{-2}\in [1/3,1/2]$ arising from the generation scheme, which shall here be noted for completeness.

The eigenvalues of $\varvec{T}$ parametrizing the synthesization model are sampled on a regular grid covering $20\times 20$ combinations of $\sigma _{1}^{-2}$ and $\sigma _{2}^{-2}$ within the specified ranges, with $\sigma _{3}^{-2}$ resulting from the given constraints. The particular coverage shown in Fig. 5 results from the generation of two orthogonally oriented linear ramps on a $20\times 20$ grid ranging from 0 to 1/3 and from 0 to 1/2 respectively, which are then complemented by a third map according to the normalization requirement. Figure 5 emerges after sorting the resulting triplets of generated eigenvalues at each point of the sampling grid in ascending order. It reveals redundancies in the coverage of eigenvalue combinations in the range $\sigma _{2}^{-2},\sigma _{3}^{-2}\in [1/3,1/2]$, which however do not conflict with the present purpose of covering the entire range of possible combinations.

## Experiment

Two simplified linear models of dark-field anisotropy parametrized by symmetric $3\times 3$ tensors (cf. Sect. 2.2) are to be compared to a physically based nonlinear reference model parametrized by an anisotropic Gaussian mass distribution likewise characterized by a symmetric $3\times 3$ tensor.

To this end, an extensive set of feasible mass distribution tensors $\varvec{T}$ (parametrizing the reference model, cf. Sect. 2.1) is generated. The range of feasible eigenvalue combinations of $\varvec{T}$ and a respective practical sampling scheme has been discussed in Sect. 2.4. For each triplet of eigenvalues (describing an anisotropic Gaussian ellipsoid), 300 rotation matrices are generated randomly (using the algorithm described in^14^), yielding a uniform distribution of eigenvectors on the unit sphere. Randomness increases the overall amount of different orientations considered over a range of eigenvalues while especially avoiding the inadvertent over- or underrepresentation of specific orientations relative to the acquisition geometry in the light of its numerous symmetries. In total, $400\times 300=120\,000$ different instances of $\varvec{T}$ are generated, covering both the feasible range of eigenvalues and orientations.

For each instance of $\varvec{T}$ describing an isolated anisotropic volume element, a set (enumerated by *i*) of $13\times 29=377$ dark-field signals $\mu _{\mathrm {DF}}^{(i)}(\varvec{T})$ is synthesized by means of Eq. (3) for a corresponding set of acquisition poses defined in Sect. 2.3. These are characterized by different combinations $(\hat{e}^{(i)},\hat{n}^{(i)})$ of the instrument’s orientations of optical axis and grating sensitivity relative to the sample coordinate system as illustrated in Fig. 4. (Note that, as already stated in Sect. 2.1, Eq. (3) is for technical reasons defined in the coordinate system of the instrument).

For each set of synthesized dark-field signals arising from an instance of $\varvec{T}$, tensors $\varvec{N}$ and $\varvec{E}$ characterizing the simplified signal models defined by Eqs. (4, 5) are reconstructed using Eq. (6) and the known acquisition geometry (defined by the set of vectors $(\hat{e}^{(i)},\hat{n}^{(i)})$ enumerated by *i*). Illustrating examples are given in Figs. 2 and 3. The relation between the simplified and the complete model of dark-field anisotropy is finally assessed by comparing their tensors’ eigenvectors and -values. An evaluation of normalized root mean square errors between the synthesized signals and the fitted simplified models provides qualitative insights into the degrees of approximation involved.

If dark-field anisotropy was actually adequately described by either of the linear models (4) or (5), three orthogonal projection trajectories (Fig. 4, blue) would fundamentally be sufficient to fully determine the respective tensor (also in a tomographic setting, cf. e.g.^10^). As such a reduced set of trajectories is highly desirable with regard to practical data acquisition, it shall therefore be considered here as well. The just described simulation experiment is therefore repeated also for a reduced set of only 3 orthogonal acquisition trajectories (marked blue in Fig. 4, yielding $3\times 29=87$ dark-field signals $\mu _{\mathrm {DF}}^{(i)}(\varvec{T})_{(3)}$).

## Results

### Goodness of fit and reproduction of dominant orientations

**Figure 6:**
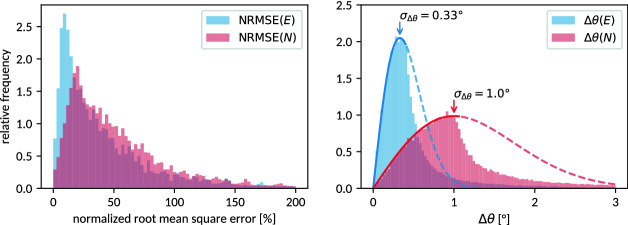
Histograms showing the normalized root mean square errors (NRMSE, cf. Eq. 8) and angular deviations (Eq. 9) of the fitted approximative tensor models with respect to the synthesized dark-field data and mass distribution tensors. The angular deviations are compared to (unnormalized) Gaussian distributions of inclination angles (integrated over the azimuthal degree of freedom, cf. Eq. 10). These are found to describe the empiric distributions up to their maximum and are used for quantification purposes.

Figure 6 (left) shows normalized root mean square errors

8 $$\begin{aligned} \text {NRMSE}=\frac{\sqrt{\overline{(\mu _{\mathrm {DF}}^{(i)}-\hat{r}^{(i)}\varvec{U}\hat{r}^{(i)})^{2}}}}{\overline{\mu _{\mathrm {DF}}^{(i)}}} \end{aligned}$$

of the linear tensor models (4) and (5) with respect to the noiseless input data $\mu _{\mathrm {DF}}^{(i)}$. Normalization of the root mean square errors puts the quantified deviations in relation to the approximated signal, thereby providing a meanigful scale. The statistical distribution of deviations arises both from the range of considered anisotropies and of considered orientations. While the distributions peak at 10-20% NRMSE, values over 100% are still encountered. These deviations reflect the highly approximative nature of the linear tensor models.

Nevertheless, the principal orientation, which is one of the central concerns of tensor tomography, is reproduced to a typical precision of $0.33{^\circ }$ and $1.0{^\circ }$ respectively for the considered reconstruction models: Figure 6 (right) compares the eigenvectors corresponding to either the smallest (in the case of $\varvec{E}$) or largest (in the case of $\varvec{N}$) eigenvalues to the original synthesized orientation of $\varvec{T}$ (cf. Fig. 3 for a visualization of the respective tensors and their extents). The considered eigenvector of each tensor indicating a volume element’s principal orientation is denoted $\hat{v}_{T}$, $\hat{v}_{E}$ and $\hat{v}_{N}$ respectively, whereby $\hat{v}_{T}$ represents the ground truth. The reconstruction error (with respect to orientation) induced by the simplified models is measured by the relative angles

9 $$\begin{aligned} \Delta \theta (\varvec{E})&=\arccos |\hat{v}_{T}\cdot {\hat{v}_{E}}| \nonumber \\ \text {and }\Delta \theta (\varvec{N})&=\arccos |\hat{v}_{T}\cdot \hat{v}_{N}|\,. \end{aligned}$$

The observed orientation error distributions are compared to a normal distribution of inclination angles, which, due to integration over the azimuthal angle, takes the form

10 $$\begin{aligned} \mathrm {PDF}(\Delta \theta ,\sigma _{\Delta \theta })\propto \sin (\Delta \theta )e^{-\frac{1}{2}\frac{\Delta \theta ^{2}}{\sigma _{\Delta \theta }}}\approx \Delta \theta e^{-\frac{1}{2}\frac{\Delta \theta ^{2}}{\sigma _{\Delta \theta }^{2}}}. \end{aligned}$$

It can be staightforwardly shown to exhibit its maximum at $\Delta \theta =\sigma _{\Delta \theta }$ in the small angle approximation $\sin (\Delta \theta )\approx \Delta \theta$. Figure 6 (right) shows to be consistent with this distribution up to the maximum value, i.e., up to its standard deviation parameter.

### Eigenvalues

**Figure 7:**
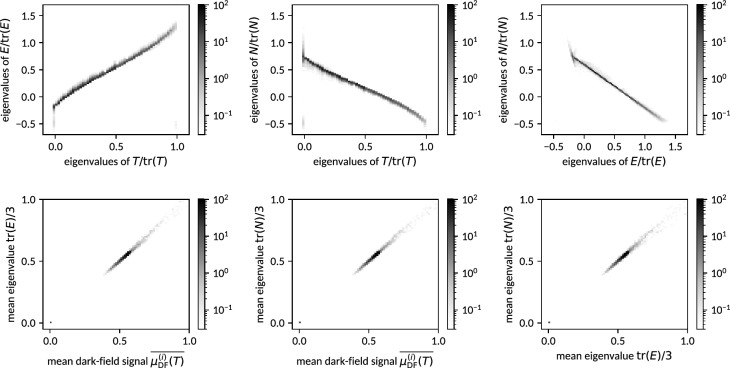
Upper row: Pairwise comparisons of the eigenspectra of tensors describing dark-field anisotropy in different ways (cf. Fig. 3). Instances of $\varvec{N}$ and $\varvec{E}$ have to this end been reconstructed from dark-field signals synthesized from generated instances of $\varvec{T}$ as detailed in Section 3. For each pair among the tensors $\varvec{T}$, $\varvec{N}$ and $\varvec{E}$ (cf. Eqs. 1–5), their respective normalized and sorted eigenvalues (in reverse order for $\varvec{N}$) are plotted against each other in 2D density histograms. Bottom row: Comparisons of mean eigenvalues of $\varvec{N}$ and $\varvec{E}$ to the mean of the dark-field signals they were reconstructed from (left and center panel), as well as comparisons among each other (right panel).

Figure 7 (top) reveals fuzzy, yet almost linear relations among the normalized and sorted eigenvalues of all models despite the strong approximations involved. Due to the expected anti-correlation of the eigenvalues of $\varvec{N}$ with those of $\varvec{T}$ and $\varvec{E}$ (cf. the illustrating example given in Figure 3), they are sorted in reverse order prior to comparison. Model (5) parametrized by $\varvec{E}$ exhibits (on average) an almost proportional relation to the original signal generating mass distribution tensor $\varvec{T}$ with moderate deviation from its positive definite nature. Model (4) parametrized by $\varvec{N}$ in contrast exhibits an inverse relation to $\varvec{T}$ with the spectrum of eigenvalues being notably shifted towards negative values. The mapping of eigenvalues nevertheless remains approximately linear as opposed to an actual reciprocal relation. The direct comparison of the normalized eigenspectra of $\varvec{N}$ and $\varvec{E}$ reveals—on average—a perfectly linear relation, indicating that both capture highly similar information given the present acquisition geometry (see Fig. 4).

The deviations from the apparent mean curves relating the different models reflect both statistical variances among different orientations of the same volume element as well as systematic deficiencies of the simplified models. As these effects are not separable in practical applications, no further effort is made to investigate their individual contributions.

Figure 7 (bottom) shows the eigenvalues’ (of both $\varvec{N}$ and $\varvec{E}$) absolute scale by means of their average values. They are found, by direct comparison, to reproduce the mean observed dark-field signal with little variance.

The case of one or two eigenvalues of $\varvec{T}$ being exactly zero corresponds to the limit of infinitely extended volume elements, causing an extreme orientation dependence and divergence of the dark-field signal according to Eq. (3) and thus also unstable (highly orientation dependent) reconstruction results in $\varvec{N}$ and $\varvec{E}$, as can be observed in Fig. 7. This limit is expected to be only of academic relevance.

### Reconstruction from three orthogonal trajectories

**Figure 8:**
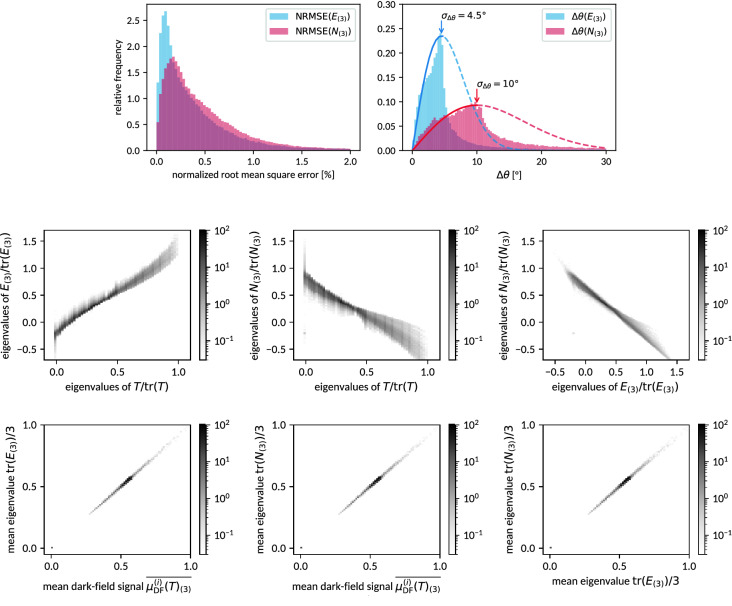
Comparison of NRMSEs, principal orientations, eigenspectra and mean signal intensities analog to Fig. 6, 7 when reconstructing tensors $\varvec{N}$ and $\varvec{E}$ from a reduced set of dark-field projections acquired along only three orthogonal projection trajectories (Fig. 4, indicated in blue).

The simulation experiment has been repeated also for a reduced set of only 3 orthogonal acquisition trajectories (marked blue in Fig. 4), which would be sufficient if either of Eqs. (4–5) were an exact description of dark-field signal anisotropy. Figure 8 depicts, analog to Figs. 6, 7, the relations between the considered models’ tensors. Despite the reduced set of data, which generally gives reason to expect fewer model inconsistencies, the observed distribution of root mean square errors is still qualitatively comparable to that found previously in Fig. 6. The eye-catching reduction of jitter within the distribution of NRMSEs as compared to Fig. 6 may however be hypothesized to be indeed related to the reduced data constraints. Although the visibly diffused relations between the tensors’ eigenspectra reflect a notably degraded relation between the reconstructed tensors and the original input, principal orientations are still roughly reproduced within an error margin of about 4.5$^{\circ }$ to 10$^{\circ }$ . Similar to the previous case, the mean eigenvalues of both $\varvec{N}$ and $\varvec{E}$ give an almost exact representation of the mean observed dark-field signal. As compared to the findings of Fig. 7 (bottom), the variance of observed values (along the diagonals) is increased though, showing that simplifying the acquisition geometry also affects the reconstruction of the signal’s zeroth order (i.e., the signal’s mean).

## Discussion

While several proofs of concept plausibly demonstrating tomographic reconstruction of sub-resolution anisotropy based on various heuristic signal models were given in previous literature, explicit validations have been lacking so far. A central assumption explicitly or implicitly shared by all current approaches is that 3D dark-field anisotropy can be described as a function of a single orientation vector. This has been, deriving from planar dark-field anisotropy, the interferometer’s direction of sensitivity. A detailed discussion of dark-field origination given recently^11^ however concludes that a complete description of general dark-field anisotropy for arbitrarily oriented structures further exhibits a non-negligible dependence also on the relative orientation of the optical axis (the direction of projection). I.e., dark-field contrast will likewise vary for anisotropic structures rotating about the axis of grating sensitivity (changing their inclination with respect to the optical axis) as for the classical case of objects rotating about the optical axis (changing their orientation with respect to the interferometer’s gratings). General dark-field anisotropy is thus fundamentally a function of two orientations: the axis of interferometer sensitivity, and the axis of projection.

With regard to tensor tomography, this observation has two remarkable implications: Foremost, it obviously raises the question why the present approaches to anisotropic dark-field tomography do nevertheless produce plausible results. And as neglecting parts of the orientation dependence is apparently tolerable, this also indicates that dark-field anisotropy may likewise be approximable as a function of the optical axis, modeling variations in scattering cross section as opposed to variations in auto-correlation properties. As both effects have opposite relations with the considered structure’s extents (cf. Fig. 2 upper right), the net relation of either model to the signal generating structure is non-obvious. The purpose of the present study therefore was to systematically investigate the actual relations based on the physically motivated signal model derived and supported experimentally previously (^11^) based on the current state of knowledge on dark-field origination.

As expected, the approximative models can exhibit large root mean square errors with regard to the actual signal, especially due to the partial neglection of orientation dependencies. Nevertheless, both linear models correctly reproduce the mean observed dark-field signal and are in particular able to recover the principal orientation of the original mass distribution up to a statistical accuracy of 0.33$^{\circ }$ to 1$^{\circ }$ when provided with dark-field data covering both orientation dependencies. Their normalized eigenvalues, which do encode the actual aspect ratios of anisotropic volume elements, are found to exhibit a fuzzy yet approximately linear relation to the normalized inverse variances of the original mass distribution (as opposed to being linear e.g. in its extents) for both models. This is the scaling behavior expected due to the influence of the volume element’s auto-correlation function along the axis of interferometer sensitivity, which appears consistent with the observation that model (5) yields slightly better reconstruction results. The eigenvalues’ absolute scale, reflecting signal intensity, does however not allow inferences on the absolute scale of the imaged structure due to the hardly separable influences of both structure scale and material density (refractive index) on the total signal strength. Further quantitative interpretations also of the observed variances in the distribution of eigenvalues are left to future research.

Although the true complexity of dark-field anisotropy (especially with regard to its orientation dependence) generally mandates a rather extensive data acquisition scheme, the consequences of using only a minimal set of three circular acquisition trajectories (which would be sufficient if the tensor models proposed for reconstruction were accurate descriptions of dark-field anisotropy) has been explicitly considered as well. While the quantitative relation between input and reconstruction is, as expected, notably degraded as compared to the preceding experiment, principal orientations for isolated volume elements could still be recovered to a statistical accuracy of 5$^{\circ }$ to 10$^{\circ }$. That these values exhibit slightly differing ratios of degradation as compared to the preceding results of 0.33$^{\circ }$ and 1$^{\circ }$ respectively is taken note of as an empiric observation. As it is conjecturable that the additional influences of noise and signal superpositions (in the tomography use case) will further challenge the stability of these reconstructions in practical applications, more comprehensive acquisition schemes as considered initially are nevertheless expected to be advisable though.

## Conclusion

The practical feasibility of dark-field tensor volume tomography depends on the applicability of linear approximations to actual dark-field signal anisotropy, given that non-linear dependencies can void the ability to solve the problem of volume reconstruction from projections. Central concern in this context was the impact of the previously unconsidered dependence of dark-field signal anisotropy also on the direction of the optical axis in addition to that of the interferometer’s direction of sensitivity, which is here the source of non-linearity and further generally challenges any models of dark-field based on functions over the unit sphere that have been assumed in all present works on non-scalar dark-field tomography. While such models must indeed be considered invalid with regard to accurate signal description and related theoretic reasoning, they can nevertheless serve the practical purpose of quantifying anisotropy properties. The present study confirms that linear tensor models modeling either of the actual orientation dependencies of dark-field contrast are indeed able to recover orientations up to a statistical accuracy on the scale of 1$^{\circ }$. These models are particularly well suited for tomographic applications, and the observed statistical variance is expected to range below other sources of uncertainty in practical applications. The observed scaling behavior of the reconstructed tensors’ eigenvalues with regard to those of the underlying mass distribution further provides first qualitative insights towards future quantitative evaluations also of aspect ratios. Central to the applicability of simplified linear models is however a comprehensive data acquisition scheme adequately addressing the actual orientation dependencies of dark-field anisotropy.
